# In vitro synthesis of tensioned synoviocyte bioscaffolds for meniscal fibrocartilage tissue engineering

**DOI:** 10.1186/1746-6148-9-242

**Published:** 2013-12-03

**Authors:** Jennifer J Warnock, Lindsay Baker, George A Ballard, Jesse Ott

**Affiliations:** 1 Clinical Sciences, College of Veterinary Medicine, Oregon State University, Corvallis, OR, USA; 2 Current address: WestVet Specialty Center, Garden City, ID, USA; 3 Current address: Peace River Veterinary Clinic, Punta Gorda, FL, USA

## Abstract

**Background:**

Meniscal injury is a common cause of lameness in the dog. Tissue engineered bioscaffolds may be a treatment option for meniscal incompetency, and ideally would possess meniscus- like extracellular matrix (ECM) and withstand meniscal tensile hoop strains. Synovium may be a useful cell source for meniscal tissue engineering because of its natural role in meniscal deficiency and its *in vitro* chondrogenic potential. The objective of this study is to compare meniscal -like extracellular matrix content of hyperconfluent synoviocyte cell sheets (“HCS”) and hyperconfluent synoviocyte sheets which have been tensioned over wire hoops (tensioned synoviocyte bioscaffolds, “TSB”) and cultured for 1 month.

**Results:**

Long term culture with tension resulted in higher GAG concentration, higher chondrogenic index, higher collagen concentration, and type II collagen immunoreactivity in TSB versus HCS. Both HCS and TSB were immunoreactive for type I collagen, however, HCS had mild, patchy intracellular immunoreactivity while TSB had diffuse moderate immunoreactivity over the entire bisocaffold. The tissue architecture was markedly different between TSB and HCS, with TSB containing collagen organized in bands and sheets. Both HCS and TSB expressed alpha smooth muscle actin and displayed active contractile behavior. Double stranded DNA content was not different between TSB and HCS, while cell viability decreased in TSB.

**Conclusions:**

Long term culture of synoviocytes with tension improved meniscal- like extra cellular matrix components, specifically, the total collagen content, including type I and II collagen, and increased GAG content relative to HCS. Future research is warranted to investigate the potential of TSB for meniscal tissue engineering.

## Background

Meniscal injuries are a common cause of painful stifle arthritis and joint dysfunction in dogs and humans [1-6]. Despite intensive research over several decades, a cure for meniscal deficiency has not been found. Thus tissue engineering is being investigated as a means for inducing meniscal healing or regeneration, through producing fibrocartilage neotissues in the laboratory, utilizing cell culture, scaffold use, and *in vitro* biomechanical stimulation.

Tissue engineering scaffolds mechanically support cell growth and guide tissue formation, and have been surgically implanted in dogs [7-9]. An ideal cell scaffold for meniscal tissue engineering and *in vivo* implantation would have physiologically relevant, meniscus like extracellular matrix (ECM) and biomechanical properties. Synthetic polymer scaffolds such as porous polyurethane [10] poly vinyl alcohol hydrogel [11], polyglycolic acid mesh [12], 75:25 poly(lactic-co-glycolic acid)[13], l-lactide ϵ-caprolactone co-polymer [14], poly(L-co-D,L-lactic acid)/poly(caprolactone-triol) [15], small intestinal submucosa [8], collagen gels [16], and pure collagen scaffolds [17,18], have been investigated for the treatment of meniscal deficiency. While these implants partially replace meniscal function, scaffolds may be complicated by progressive chemical breakdown, structural weakening, lack of lubricity, inadequate integration with the recipients’ tissues [19], articular cartilage contact damage [20] and inflammatory reactions [18] incited by allogenic or xenogenic scaffold content [21].

As harvest of autologous meniscal cells would cause patient injury, this study used canine osteoarthritic, autogenous joint- origin synoviocytes obtained from arthroscopic debris collected during clinically indicated stifle surgery. To avoid additional patient morbidity, synoviocytes from normal, unaffected joints were not utilized; additionally, normal canine synovium has been found to have less chondrogenic potential compared to infrapatellar fat pad [22]. In rats, mice, and humans, synovium is superior to other cell sources, including periosteum, bone marrow, muscle, and adipose, for in vitro chondrogenesis [23-25]. In the dog, osteoarthritic joint-origin synovial cells can produce components of meniscal fibrocartilage *in vitro*[26,27] and are readily obtained minimally invasively [28]. In vivo, canine osteoarthritic joint- origin synoviocytes are responsible for cellular repopulation of meniscal grafts [29] and undergo spontaneous fibrochondrogenesis [30], including formation of a meniscal-like regenerate in meniscectomized stifles [31]. Synovial pedicle grafts and free synovial grafts have been used to achieve partial avascular meniscal healing in dogs and rabbits [32-35] and are superior to muscle flaps and synthetic meshes in dogs [33]. Use of living, autologous bioscaffolds with these regenerative properties could be advantageous over use of synthetic scaffolds for augmenting meniscal healing or treating meniscal loss.

Recently, scaffold-free tissue-engineered constructs cultured from synovial mesenchymal stem cells were used to successfully induce healing of meniscal defects [36] in pigs. Thus, given this success in the pig, and to avoid the complications of scaffold use, the objective of this study is to produce autologous fibrocartilage bioscaffolds, which in the future may be utilized in the dog as surgical implants without synthetic, xenogenic, or allogenic components. We hypothesize that the long term application of tension to synoviocytes will result in formation of a collagenous bioscaffold, with greater cell viability, and increased meniscal-like extracellular matrix content and architecture, versus synoviocytes grown short term in hyperconfluent monolayer culture.

## Methods

### Tissue harvest

With informed owner consent, synovium was obtained from 10 dogs with naturally occurring clinical osteoarthritis as per institutional Animal Care and Use Committee Protocol. Dogs were treated for degeneration of the cranial cruciate ligament and medial meniscal injury via exploratory arthroscopy, partial meniscectomy if indicated, and tibial plateau leveling osteotomy or lateral tibiofabellar suture. Synovial villi were arthroscopically harvested during routine partial synovectomy, only as clinically required, using a tissue shaver (Stryker, San Jose, CA) with a 3.5 mm aggressive shaver blade run at 1800 rpm [28]. Dogs with a history of steroid administration, or dogs with concurrent disease processes were excluded from the study.

Harvested synovial villi were immediately placed in a 50 mL polypropylene tube containing 40 mL of Dulbeccos’ Modified Eagle’s Media (DMEM) with 10% fetal bovine serum (FBS), warmed to 37°C. The tube was then transported immediately to the laboratory and centrifuged at 313 g, media was decanted, and resultant tissue pellet weighed, and transferred by pipette to a digestion solution as described below.

### Cell culture

Tissue fragments were completely digested with sterile Type 1A clostridial collagenase 10 mg/mL in RPMI 1640 solution over 2–6 hours at 37°C. Tissue was deemed to be completely digested when no ECM could be visualized grossly. Cells were cultured in monolayer for 4 passages to isolate Type B fibroblast-like synoviocytes [37] and Type C intermediate synoviocytes [37]. The following media formulation was used for the duration of culture: high glucose DMEM with phenol red pH indicator, supplemented with 17.7% FBS, 0.021 mg/mL glycine, 0.025 mg/mL L-alanine, 0.037 mg/mL L- asparagine, 0.038 mg/mL L-aspartic acid, 0.042 mg/mL L-glutamic acid, 0.033 mg/mL L-proline, 0.030 mg/mL L-serine, 0.23 mg/mL pyruvate, 0.52 mg/mL L-glutamine, 6.75 mg/mL HEPES buffer, 177.0 units/mL penicillin, 177.0 μg/mL streptomycin, and 0.44 ug/mL amphoterocin (supplemented DMEM, “sDMEM”). The flasks were incubated at canine body temperature, at 37.8°C, and at 5% CO_2_, 95% humidity, with sterile media change performed every 24 hours.

**Figure 1:**
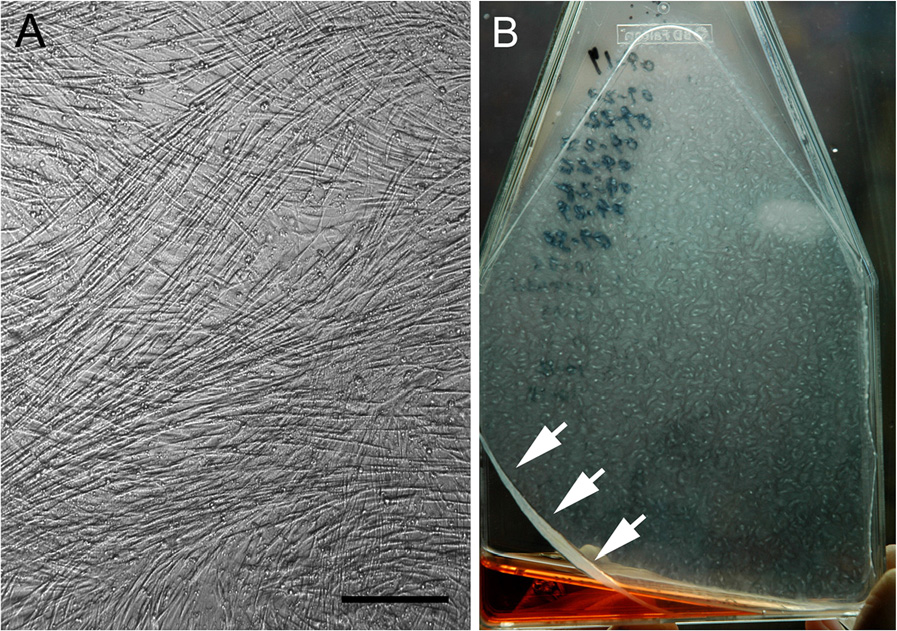
**Representative hyperconfluent cell sheets: Phase contrast microscopy of 4**^**th **^**passage hyperconfluent cell sheet (“HCS”; A), 10X objective magnification, bar = 100 μm.** Gross appearance of a representative hyperconfluent 4^th^ passage cells sheet at commencement of spontaneous sheet contraction (arrows), indicating time for sheet harvest to synthesize tensioned synoviocyte bioscaffolds **(B)**.

For each dog, synoviocytes were passaged when 95% monolayer confluence was reached. For all dogs at 4^th^ passage a mean of 6,300,000 ±10,000 cells per flask were seeded into eighteen 150 cm^2^ flasks. These 4^th^ passage cells were allowed to become hyperconfluent monolayer cell sheets (“HCS”), defined as cells overlapping each other in greater than 100% confluency (Figure 1A). When HCS began to spontaneously contract off the corners of the flask floor (Figure 1B), they were completely dislodged off the flask floors by gentle pushing with the pipette tip. At this time 3 HCS per dog were harvested for tissue analyses (as described below), 6 were required for use in another unrelated study, and the remaining 9 were used to make tensioned synoviocyte bioscaffolds (TSB).

**Figure 2:**
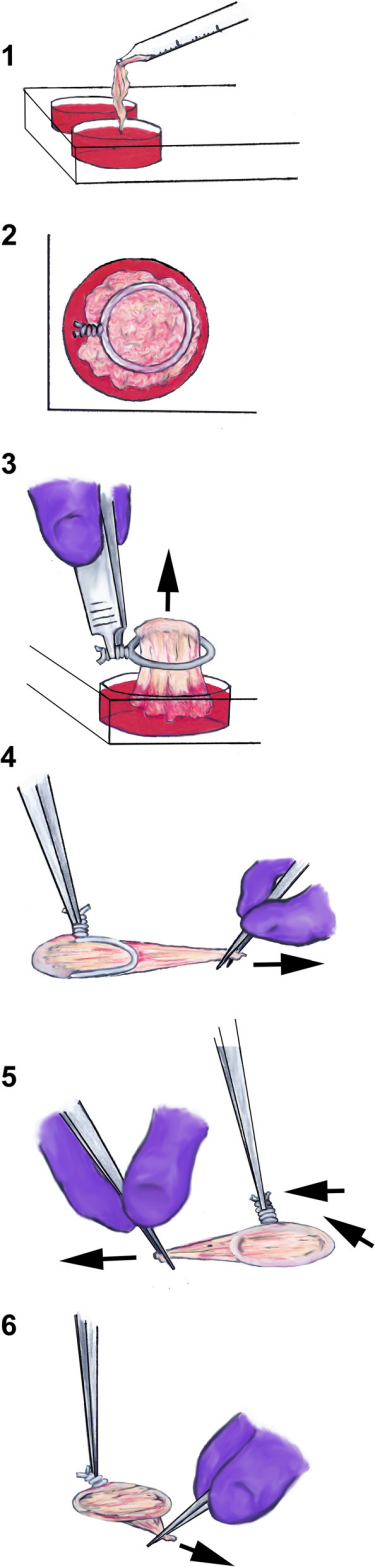
Illustration of tensioned synoviocyte bioscaffold synthesis: 1) the hyperconfluent cell sheet (“HCS”) is moved to the cell culture well using a pipette tip; 2) the wire hoop is placed over the HCS; 3) while holding the wire twist with a forceps, the HCS is doubled over one end of the wire hoop; 4) the ends of the doubled over HCS are grasped with forceps and tension is applied perpendicular to the wire hoop; 5) the HCS is wrapped over the opposite side of the wire hoop; 6) the triple wrap is finished, creating a tensioned synoviocyte bioscaffold with six cell layers.

To synthesize TSB (Figure 2), each HCS was moved from the flask to a 6-well plate without touching the flask nozzle, using a 10 mL pipettor and gentle continuous suction. The HCS was gently discharged into a 10 mL media well, filled with the above described media to prevent cell sheet desiccation. A 2.0 cm diameter 22ga wire hoop was placed over the cell sheet. While grasping the twist on the wire hoop with a tissue forceps, the hyperconfluent cell sheet was pulled around the hoop using Bishop Harmon forceps. The hypercondfluent cell sheet was pulled over the hoop three times with approximately 0.5 N of tension to avoid tearing, making a tensioned bioscaffold. Then the completed TSB was placed on the bottom of the well with the free end down to prevent unraveling. TSB were cultured for an additional 30 days with daily media changes and then harvested for tissue analyses as described below.

### Tissue analyses

Tissue analyses of HCS and TSB examined presence of ECM which is functionally critical in the normal meniscus: type I collagen which accounts for the preponderance of meniscal collagen [38]; type II collagen, which accounts for a small amount primarily localized to the axial meniscus, [38] α- smooth muscle actin (ASM), [39-41]; and glycosaminoglycans (GAG) [42-44], including aggrecan [45].

### Cell viability

During monolayer culture, cell viability counts were performed using the trypan blue exclusion assay [46] at each passage on all dogs. One HCS each from 3 dogs and one TSB each from all dogs were washed three times in sterile phosphate buffered saline and immersed in 4 μM ethidium homodimer and 6 μM acetomethoxy calcein (calcein –AM) solution (Ethidium homodimer and Calcein AM Live/ Dead Viability Assay, Invitrogen, Carlsbad, CA) for 20 minutes at 37.8°C, 5% CO2, 95% humidity. Live and dead cell counts were performed by hand on 6 regions (3 of the periphery and 3 of the center) of each construct, where cells could be clearly visualized in one plane, using a laser microscope (Eclipse Ti-u Laser Microscope, Nikkon, Japan). Due to the complex three –dimensional nature of the neotissues, these cell counts provided an estimate of cell viability.

### Histology

Formalin-fixed and paraffin embedded sections of one HCS and two TSB per dog were stained with Hematoxylin and Eosin, Masson’s trichrome, and Toluidine Blue.

For immunohistochemistry, tissues were sectioned at 4–5 μm and sections were collected on charged slides and baked at 60°C for 1 hour. Slides were rehydrated through two washes of xylene, two washes of 100% ethanol, and one wash of 80% ethanol and water. Slides to be stained for collagen were pretreated with a pepsin digestion of 0.1% pepsin in 0.1 M HCl at 37°C for 5 minutes. All slides were rinsed in Tris buffered saline (TBS), placed on an autostainer and washed in TBS followed by 3% H_2_O_2_ in TBS for 5 minutes. Then serum-free protein block (Serum Free Protein Block, Dako, Carpinteria CA, #X0909) was applied for 10 minutes, with excess blown off. The primary antibodies were diluted in a proprietary antibody diluent (Antibody Diluent, Dako, Carpinteria, CA) to the following concentrations: Collagen I (rabbit Collagen Type I Antibody (#AB749P), Millipore, Temecula, CA) 1:100, Collagen II (rabbit Collagen Type II Antibody (#AB746P), Millipore, Temecula, CA), 1:100; alpha smooth muscle actin (mouse Alpha Smooth Muscle Actin Antibody (#M0851), Dako, Carpinteria, CA)1:30; and mouse or rabbit universal negative control antibodies (Universal Negative Control, Dako, Carpinteria, CA) were used to test for non-specific immunoreactivity. Antibodies were applied to experimental neotissues or control tissues for 30 minutes at room temperature. Positive control tissues (Envision + HRP, Dako, Carpinteria, CA) type II collagen included canine articular cartilage, meniscus, and tracheal cartilage; for type I collagen, meniscus, tendon, and skin; small intestine for α- smooth muscle actin; and lymph node for macrophages. After washing in TBS, secondary antibodies were applied for 30 minutes at room temperature, then washed again with TBS. The chromogen (Nova Red, Vector Laboratories, Burlingame, CA) was applied for 5 minutes as directed by the manufacturer, and washed in deionized water followed by hematoxylin for 5 minutes, rinsed again in deionized water, then rinsed in TBS, and coverslipped.

Histologic specimens were examined completely at 10x and 20x objective magnification (Zeiss Microscope, Thornwood, NY) and images of each section were captured by a digital camera (Olympus DP-70 Digital Camera, Olympus, Melville, NY). Immunoreactivity staining intensity for each was rated as originally described by Wakshlag et al. [47] with a few modifications: immunoreactivity was localized to intracellular or extracellular staining and intensity was described as weak, moderate, and strong staining. Intracellular and extracellular immunoreactivity was described as being rare if <10% of the cells or ECM area was positively stained, patchy if 10-50% of cells or ECM were stained, and diffuse if >50% of cells or ECM was stained.

### Tissue weight

One HCS and one TSB per dog were lyophilized and a dry weight obtained. Samples were digested in 1.0 ml Papain Solution (2 mM Dithiothreitol and 300ug/ml Papain) at 60°C in a water bath for 24 hours. This papain digest solution was used to obtain double stranded DNA (dsDNA), GAG, and collagen content of the HCS and TSB.

### DNA quantification

Double stranded DNA quantification assay (The Quant-iT PicoGreen™ Assay, Invitrogen Carlsbad, CA) was performed per manufacturer’s instructions; double stranded DNA extracted from bovine thymus was used to create standards of 1,000, 100, 10, and 1 ng/mL. Standard and sample fluorescence was read by a fluorometer (Qubit, Invitrogen, Carlsbad CA) at 485 nm excitation/ 528 nm emission and dsDNA was determined based on the standard curve.

### Biochemical extracellular matrix assays

Glycosaminglycan content was determined by the di-methyl-methylene blue sulfated glycosaminoglycan assay [48] using a spectrophotometer (Synergy HT– KC4 Spectrophotometric Plate Reader and FT4software, BioTec, Winooski, VT). The Chondrogenic Index was calculated using the following equation: [μg GAG/ ug dsDNA] [49] and [μg collagen/ ug dsDNA] to identify chondrogenic cellular activity of each tested culture type. Collagen content was determined by Erlich’s hydroxyproline assay, as described by Reddy et al. [50] Hydroxyproline content was converted to collagen content using the equation: [μg hydroxyproline × dilution factor/ 0.13 = μg collagen] (Ignat’eva et al. [51]), because hydroxyproline is approximately 13% of the amino acids in human meniscal collagen [52]. Collagen Index was calculated using the following equation: [μg collagen/ ug dsDNA] to determine cellular collagen production. GAG and collagen content were also standardized to tissue dry weight and expressed as% dry weight [53].

### Statistical methods

Data was analyzed using a paired 2-tailed Student’s t-test using statistical software (Graphpad Prism, La Jolla, CA). All data is reported as mean ± Standard Error of the Mean (SEM). Statistical significance was declared at *P* ≤ 0.05.

## Results

The mean age of dogs was 5.4 years, (range 2–8 years). Breeds represented included: Labrador Retriever (3), Boston Bull Terrier, Australian Shepherd, Rottweiler (2), Doberman Pincer, Labrador cross, and mixed breed; 5 dogs were male neutered, 4 dogs were female spayed, and 1 was an intact female. As observed by a Diplomate of the American College of Veterinary Surgeons –Small Animal, all dogs had synovitis and osteophytosis, and grade 1–2 Outerbridge cartilage lesions [54].

### Cell culture, cell viability, and cellularity

Cell culture: The mean wet weight of harvested synovium was 1.59 g, (range: 0.15 g- 4.8 g). As found previously [28], the preponderance of cells harvested arthroscopically were red blood cells. A mean of 2.27 × 10^6^ ± 6.6 × 10^5^ synoviocytes per dog were obtained at harvest. Time from plating at 4^th^ passage to hyperconfluency and commencement of spontaneous contraction off the plate floor was a median of 5 days (range 2.3 -8 days).

**Figure 3:**
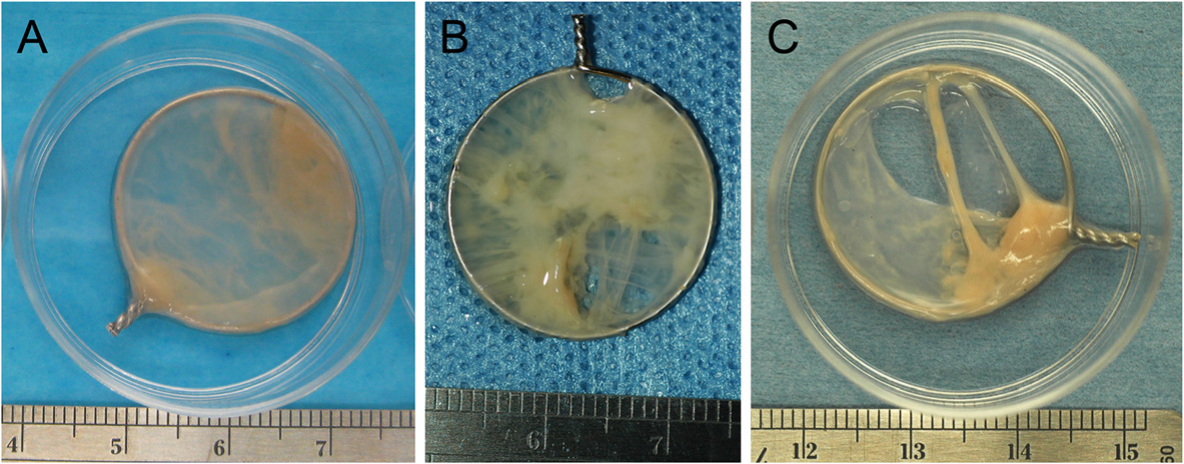
Tensioned synoviocyte bioscaffolds: Gross appearance of two representative tensioned synoviocyte bioscaffolds, (“TSB”; A and B); a TSB that contracted itself apart to form an incomplete TSB (C).

Timing of TSB synthesis was critical and needed to be performed upon first observation of cell sheet contraction as described above. Failure to immediately harvest HCS resulted in the formation of contracted masses. By the end of the 1st week of culture, TSB were able to be moved without unraveling. Long term culture with tension resulted in a sheet- like appearance of TSB, with some TSB appearing more translucent (Figure 3A) while others appeared more opaque (Figure 3B). Of the 9 attempted TSB made per dog, an average of 2.3 TSB per dog partially contracted off their wire hoops, forming an incomplete sheet or contracted mass, (Figure 3C) and were not analyzed in this study. One dog, a 7 year old male neutered Doberman pincer, produced hyperconfluent cell sheets that were thin and fragile and disintegrated upon manipulation, precluding formation of TSB in this individual. For the first 2 weeks after TSB were synthesized, the culture well media had become light orange by the time the daily media change was due, indicating a drop in pH.

Viability: For monolayer culture cell viability was 99% for passages 1–3. Mean cell viability of 4^th^ passage monolayer cells was 98.8% ±0.4 versus passages 1–3 (P = 0.78). Mean cell viability of HCS was 93.3% ±1.7 compared to 4^th^ passage cells (P = 0.04). TSB viability cell counts represented an estimation due to their 3 –dimensional structure. Cell viability of TSB was 74.9% ±5.6, representing a decrease from 4^th^ passage cells and HCS (P = 0.005 and P = 0.01, respectively).

Double Stranded DNA Content: Percent tissue DNA content standardized to dry weight was 0.18% ± 0.0002 for HCS and 0.20% ± 0.0003 for TSB (P = 0.5494).

**Figure 4:**
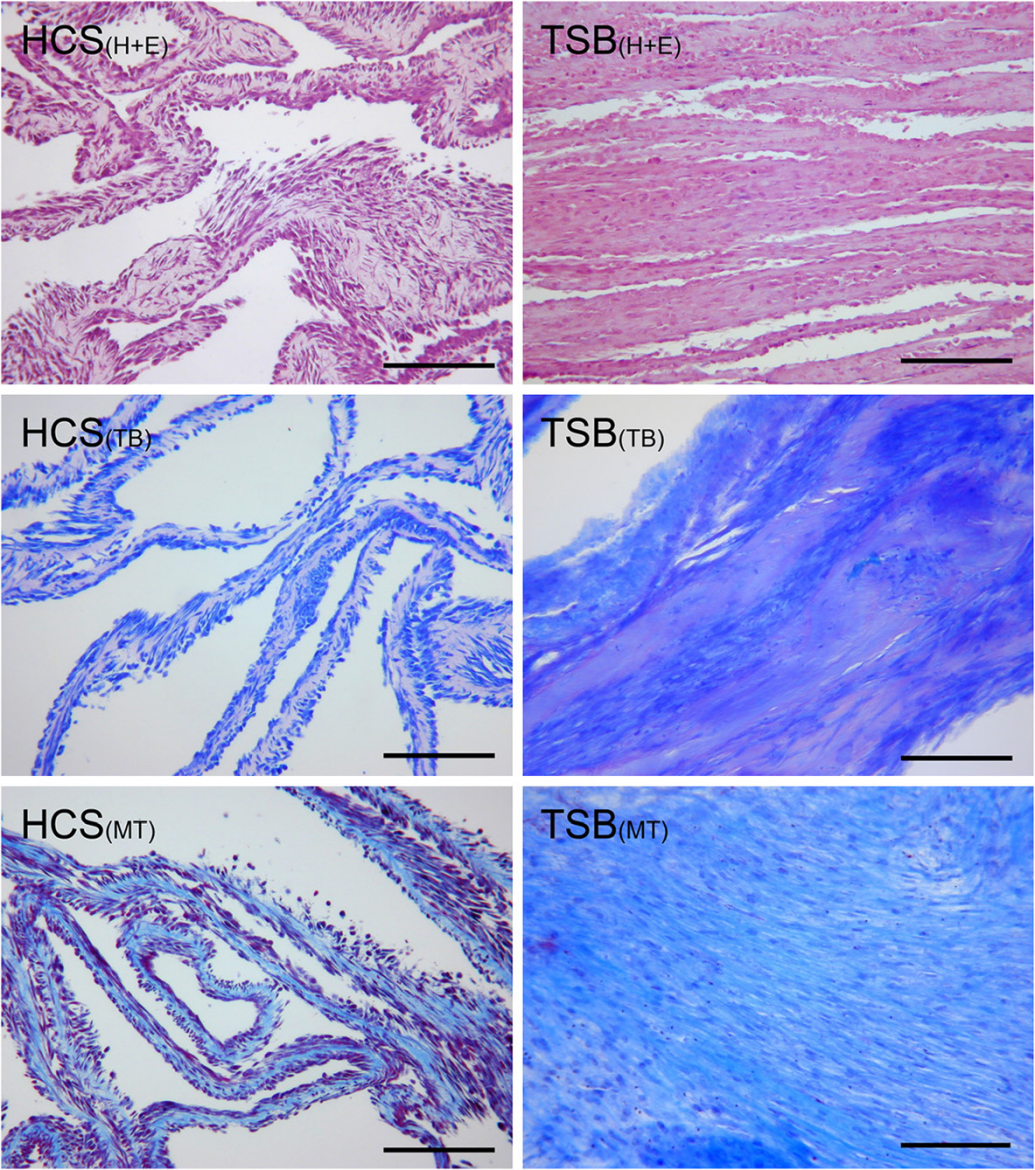
**Histologic Analysis: Hematoxylin and Eosin stain (“H + E”), Toluidine Blue stain for GAG (“TB”), and Masson’s Trichrome stain for collagen (“MT”) of hyperconfluent cell sheets (“HCS”) and tensioned synoviocyte bisocaffolds (“TSB”).** 10X objective magnification, bar = 100 μm. Note the difference in tissue architecture between the thin HCS and dense bands and sheets of extracellular matrix in TSB.

Hematoxalin and Eosin Histologic Analysis: HCS contained fibroblastic type cells with light, homogenous, eosinophilic ECM. TSB had dense ECM organized in bands and sheets. TSB cells appeared round to fusiform with the long axis parallel to the vector of tension (Figure 4).

### Glycosaminoglycan content

Dimethylmethylene Blue (DMMB) Assay: Percent GAG per dry weight was 1% ± 0.0005 for HCS and 1.8% ±0.001 for TSB (P = 0.001). On a cellular level, TSB synoviocytes produced more GAG per cell (per the Chondrogenic Index), at 10.7 ± 1.4 versus 6.2 ±0.7 for HCS (P = 0.0052).

Toluidine Blue Histologic Analysis: All HCS had light homogenous GAG deposition in between cell layers. TSB contained regional GAG deposition (Figure 3). In TSB from 2 dogs, some of the cells within GAG deposits were rounded in shape and were located in pseudo lacunae, similar to cells of the axial meniscus or articular cartilage.

### Collagen content

Hydroxyproline Assay: TSB had higher collagen content at 13.1% ±0.02 versus the 5.7% ±0.01 of HCS (P = 0.0137). There was a trend that TSB synoviocytes produced more collagen per cell (per the Collagen Index), at 78.1 ±19.4 versus HCS at 34.7 ±7.1 (P = 0.05).

Trichrome Blue Histologic Analysis: Hyperconfluent monolayer synoviocyte sheets contained light collagen accumulation between cell layers. TSB contained dense collagen bands and sheets with cells oriented parallel to the vector of tension (Figure 4).

**Figure 5:**
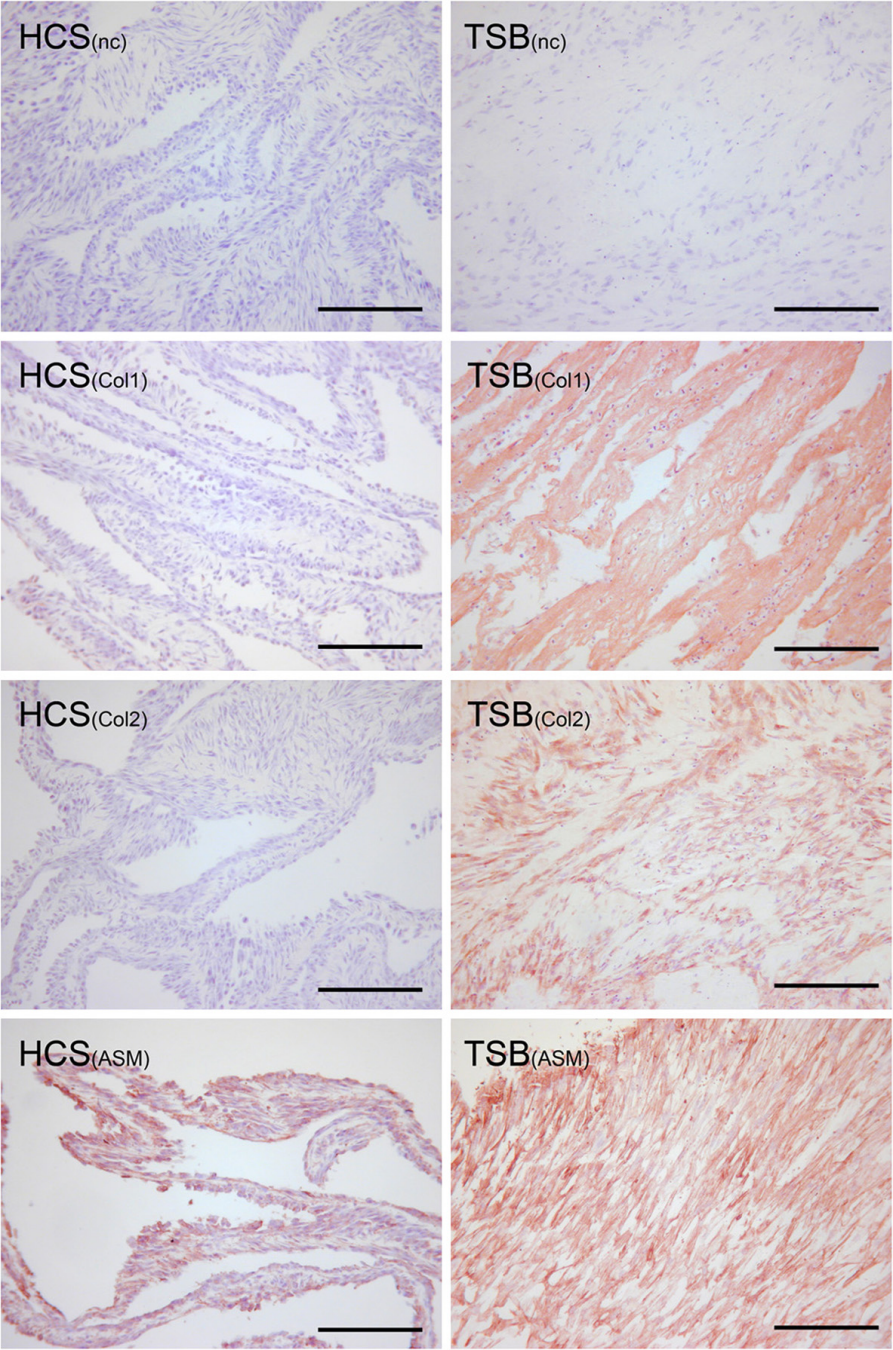
**Immunohistochemistry analysis: Immunohistochemistry for collagens type I and II (“Col1 and Col2”), and alpha smooth muscle actin (“ASM”), of hyperconfluent cell sheets (“HCS”) and tensioned synoviocyte bisocaffolds (“TSB”).** Immunohistochemistry negative controls are delineated by “NC.” 10X objective magnification, bar = 100 μm; Nova Red chromogen. In this example, TSB has moderate extracellular matrix staining for type I collagen, moderate intracellular immunoreactivity to type II collagen, and strong intracellular immunoreactivity to alpha smooth muscle actin.

Collagen Immunohistochemistry: Mild patchy intracellular immunoreactivity to type I collagen was observed in all HCS. TSB 8 of 9 dogs had moderate to strong type I intracellular immunoreactivity over 50% of the cell population, with diffuse moderate immunoreactivity over the entire bioscaffold (Figure 5). One dog, a 7.3 year old female spayed mixed breed had mild intracellular and moderate extracellular immunoreactivity over 50% of the cell population and ECM area.

**Figure 6:**
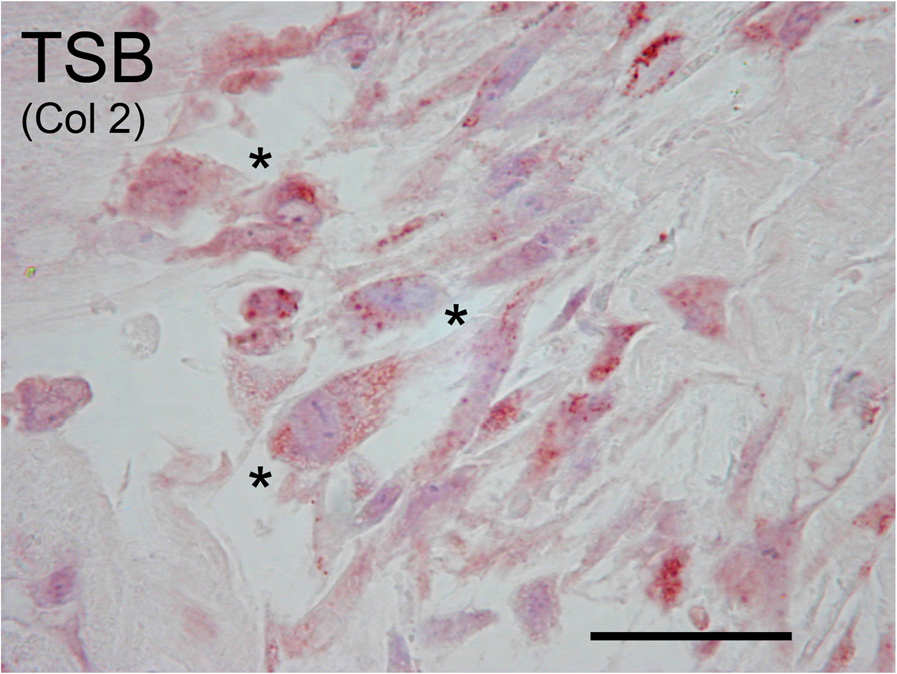
Evidence of mesenchymal progenitor cells: Immunohistochemistry for collagen type II in a tensioned synoviocyte bioscaffold (“TSB Col2”), showing variably shaped, large, fibroblastic cells (denoted by *) with moderate intracellular immunoreactivity to type II collagen; these cells are likely synovial mesenchymal progenitor cells. 20X objective magnification, bar = 50 μm; Nova Red chromagen.

All HCS were negative for type II collagen immunoreactivity. In 7 dogs TSB had moderate to strong intracellular type II collagen immunoreactivity in 10-50% of cells while 1 dog had mild immunoreactivity in 10% of cells (Figures 5). When examined at higher magnification these positively staining cells were grouped in clusters and had variable size and shape (Figure 6). TSB contained mild immunoreactivity to type II collagen over 10-50% of the bioscaffold ECM in 4 dogs and less than 10% in 3 dogs. One dog, an 8 year old male neutered Labrador, was negative for ECM type II collagen immunoreactivity, and the above listed 7.3 year old female spayed Labrador was also negative for intracellular and ECM type II collagen immunoreactivity.

### Alpha smooth muscle actin content

Strong intracellular immunoreactivity to ASM was expressed in all HCS and TSB (Figure 5).

## Discussion

Of the many synthetic and biologic materials available to create scaffolds for meniscal tissue engineering, use of type I collagen has the closest functional significance. Type I collagen is the principal functional ECM component of the menisci, which is organized into circumferential bands to convert weight bearing forces into tensile hoop strains [52,55,56]. Because tension is a principle biomechanical stimulus for type I collagen formation *in vivo*[38,57,58] and *in vitro*[59,60], tension was utilized in the present study with the goal of producing a bioscaffold that is rich in type I collagen. Synoviocytes are mechanosensitive to tension [61,62]; synoviocytes cultured in monolayer at 50-60% confluence and exposed to static tension increase hyaluronic acid production by 57% [61]. In the present study long term culture with tension increased collagen content relative to HCS Long term culture with tension also produced tissue architecture consisting of bands and sheets of collagen, with longitudinally oriented cells, which is closer to the histologic appearance of the meniscus versus the architecture of HCS. This is the first report that static tension over long term culture can increase collagen content towards a fibrocartilage tissue type in synovial fibroblasts.

The concentration of collagen in normal rabbit synovium per dry weight is approximately 11% [63], which is slightly lower than the 13% collagen of TSB, and higher than the 5% of HCS. The lower collagen content of HCS may reflect the diseased origin of our synovial tissues or may be a result of the artificial monolayer culture environment. At this time the ideal collagen content of meniscal implants is not known, but maximizing collagen content would likely improve the strength and durability of a surgical implant.

GAG and type II collagen are major ECM synthesis targets in meniscal tissue engineering because they are functionally critical components of the meniscus, particularly in the non- healing, axial, avascular zone [38,45]. In the present study TSB were 1.8% GAG, and as observed histologically, the majority of TSB contained type II collagen. In contrast, native synovium produces collagen types I,III, and VI [64], and contains only 0.7% of GAG per dry weight [63]. In this study, regional deposition of GAG and type II collagen were synthetic products of mesenchymal synoviocyte progenitor cells [22] (Figure 6), the numbers of which were increased by the high concentration of FBS and long term culture [65,66]. The variation seen in type II collagen formation between dogs may have been due to variable numbers of mesenchymal progenitor cells per dog, and their varying differentiation potential within each individual [67]. Another potential mechanism for GAG and type II collagen deposition involves biomechanical stimulation. Type II collagen and GAG form when synoviocytes are exposed to compressive loads in vitro [68], which may have been generated in the TSB during tensioning due to Poisson’s effect. Poisson’s effect states that when a material is tensioned, it contracts transversely to the vector of pull (which can be observed when stretching a rubber band, for example), thereby forming regions of compression within the material. In addition, FBS does contain chondrogenic growth factors, which support joint growth of the fetal calf in utero; however the quantity and proportion of these factors were not determined in this study.

In meniscal development, the undifferentiated meniscal primordia are highly cellular with minimal ECM [69], which mature into fibrocartilage with few cells and dense ECM. Because tissue differentiation is highly dependent on cell density [70], we attempted to recapitulate this developmental process with long term culture of highly cellular HCS tensioned as TSB. While ECM did increase in TSB (Figure 3), total dsDNA concentration, as a measure of tissue cellularity, was not different between HCS and TSB. Further, cell viability dropped over time, possibly due to apoptosis resulting from prolonged cell culture. Additionally, culture of highly cellular TSB in 9 mL of media with once daily media changes limited nutrient delivery and resulted in a daily pH drop, contributing to cell mortality.

Canine synovium expresses alpha smooth muscle actin [71]. In the present study, the presence of ASM in HCS and TSB explains the contractile behavior of HCS, maintenance of tension of TSB, as well as contraction of some TSB off their wire hoops. This contractile behavior of a bioscaffold implant could be utilized to assist in apposition of meniscal wound edges for augmentation of meniscal healing.

In prior research, we found that culture of canine synoviocytes as HCS in monolayer over a 30 day period resulted in spontaneously contracted masses with poor cell viability, poor ECM formation, and poor handling characteristics [28]. However, in the present study, we did not repeat long term monolayer culture as a control to TSB, which is a weakness in our study.

## Conclusions

In conclusion, we partially accept our hypothesis, as addition of tension over longer term culture in TSB resulted in higher GAG and collagen content, versus HCS. DNA content was not different between TSB and HCS. However, cell viability dropped over time in TSB. Thus, TSB is a viable model for future *in vitro* meniscal tissue engineering studies, but further investigation is required to reduce TSB cell mortality, increase collagen content, and reduce inter-patient variability of these constructs.

## Abbreviations

ASM: Alpha smooth muscle actin; Calcein –AM: Acetomethoxy- calcein; DMEM: Dulbecco’s modified Eagle’s media; hgDMEM: High glucose Dulbecco’s modified Eagle’s media; sDMEM: Supplemented Dulbecco’s modified Eagle’s media; ECM: Extracellular matrix; FBS: Fetal bovine serum; GAG: Glycosaminoglycans; HCS: Hyperconfluent monolayer cell sheet; IHC: Immunohistochemistry; SEM: Standard error of the mean; TBS: Tris buffered saline; TSB: Tensioned synoviocyte bioscaffold.

## Competing interests

The authors declare that they have no competing interests.

## Authors’ contributions

JW conceived of and designed the study, participated in cell culture and data acquisition, and drafted the manuscript. LB and GB were DVM students of the College of Veterinary Medicine at the time of this study, and carried out cell culture, tissue analysis assays, and helped with study design. JO organized and performed assays and coordindated study participants. All authors read and approved the final manuscript.
